# Home Blood Pressure Monitoring: New Evidence for an Expanded Role

**DOI:** 10.1371/journal.pmed.1001592

**Published:** 2014-01-21

**Authors:** Mark Caulfield

**Affiliations:** Barts and The London School of Medicine and Dentistry, Queen Mary University of London, London, United Kingdom

## Abstract

In a Perspective, Mark Caulfield discusses potential implications of using home blood pressure monitoring for diagnosis and treatment of hypertension.

*Please see later in the article for the Editors' Summary*

**Linked Research Article**

This Perspective discusses the following new study published in *PLOS Medicine*:

Asayama K, Thijs L, Brguljan-Hitij J, Niiranen TJ, Hozawa A, et al. (2014) Risk Stratification by Self-Measured Home Blood Pressure across Categories of Conventional Blood Pressure: A Participant-Level Meta-Analysis PLoS Med 11(1): e1001591. doi:10.1371/journal.pmed.1001591

Jan Staessen and colleagues compare the risk of cardiovascular, cardiac, or cerebrovascular events in patients with normal office blood pressure but elevated home blood pressure.

Cardiovascular disease is now the leading cause of death and disability worldwide [1]. The application of electronic blood pressure measurement (home or ambulatory monitoring) has been shown to improve the precision of diagnosis of hypertension and is superior to conventional, or clinic, blood pressure monitoring at predicting prognosis in those with high blood pressure. The global burden of hypertension now affects over 1 billion people and contributes to 80% of cardiovascular disease outcomes in emergent economies [1]. From observational studies of blood pressure (mostly clinic blood pressure) in 1 million people, for every 20-mm Hg increment in systolic blood pressure greater than 115 mm Hg, there is an effective doubling of cardiovascular mortality [2].

## The Prognostic Role of Electronic Blood Pressure

There are limited data on the use of home blood pressure monitoring (HBPM) to assess the cardiovascular risk of patients. An analysis as part of the 2011 UK National Institute for Health and Care Excellence guideline for hypertension suggested that ambulatory blood pressure monitoring (ABPM) was superior to HBPM and that both were superior to clinic blood pressure monitoring (CBPM) as a guide to adverse outcomes [3],[4]. This analysis led to the recommendation that ABPM be used to confirm a diagnosis when hypertension is suspected, but the panel (in which I was a participant) acknowledged that the relative lack of data on HBPM might have affected prognostic accuracy [3],[4].

As published in this week's *PLOS Medicine*, Jan Staessen and colleagues undertook an individual-patient meta-analysis based on data from the International Database of Home Blood Pressure in Relation to Cardiovascular Outcome. The meta-analysis included 5,008 people who had home and conventional blood pressure measurements and were not being treated with antihypertensive medications that would have influenced prognostic outcomes [5]. These measurements were used to stratify participants into five categories of blood pressure: optimal, <120/<80 mm Hg; normal, 120–129/80–84 mm Hg; high-normal, 130–139/85–89 mm Hg; mild hypertension, 140–159/90–99 mm Hg; and severe hypertension, ≥160/≥100 mm Hg.

## Home Blood Pressure Monitoring Improves Risk Stratification

In keeping with a previous analysis, the meta-analysis found no significant improvement in risk stratification in those defined as severely hypertensive (≥160/≥100 mm Hg); at these levels HBPM and CBPM are both strong predictors of outcomes. This is not unexpected; severe hypertension does not lack precision in risk stratification and is not difficult to decide to treat. On the other hand, at every level of blood pressure below severe hypertension, the additional measurements obtained from HBPM improved risk stratification, providing new evidence supporting the use of HBPM in routine assessment of risk. This result is important because it could refine risk stratification in people with optimal, normal, or high-normal blood pressure based on CBPM, who are not conventionally treated. In addition, HBPM showed improved stratification of risk in those with masked hypertension, that is, those who have normal clinic blood pressure but on HBPM or ABPM have periods of elevated blood pressure and may benefit from treatment [5].

These findings add depth to the evidence base in favour of electronic blood pressure monitoring in the form of HBPM. However, the authors do not have data to provide a head-to-head comparison of HBPM and ABPM, which would be valuable in assessing whether HBPM could be of sufficient diagnostic and prognostic precision to replace ABPM in the confirmation of a diagnosis informing a decision to treat. In addition, they were not able to standardise HBPM approaches, but that limitation would be more likely to dilute the observed improved risk stratification by HBPM than create a spurious association. To address this issue and validate these findings, the authors suggest further comparative, prospective randomised controlled trials would be valuable [5].

## The Potential Implications of These Findings for Patients with High Blood Pressure

As the authors suggest, the use of electronic blood pressure monitoring (HBPM and ABPM) is likely cost-effective, allows more rapid diagnosis and treatment, saves consultation time, and may in some people avert treatment at least temporarily [6]. In this study by Staessen and colleagues, HBPM appears valuable in assessing those at risk who would not usually be considered as potentially benefiting from treatment. With a growing burden of high blood pressure and a growing availability of affordable devices, HBPM could be used to diagnose high blood pressure and help decide whom to treat. It empowers patients to take on a role in assessment of their blood pressure. Now, with smart phone applications that accept automated data uploads from HBPM and display blood pressure trends over time, HBPM could help avoid travel and may save time for the health care team as they conduct remote consultations exploiting electronic tools for communication.

## Abbreviations

ABPM: ambulatory blood pressure monitoring
CBPM: clinic blood pressure monitoring
HBPM: home blood pressure monitoring
